# UK research data resources based on primary care electronic health records: review and summary for potential users

**DOI:** 10.3399/BJGPO.2023.0057

**Published:** 2023-08-09

**Authors:** Lara Edwards, James Pickett, Darren M Ashcroft, Hajira Dambha-Miller, Azeem Majeed, Christian Mallen, Irene Petersen, Nadeem Qureshi, Tjeerd van Staa, Gary Abel, Chris Carvalho, Rachel Denholm, Evangelos Kontopantelis, Ayoyemi Macaulay, John Macleod

**Affiliations:** 1 Health Data Research UK (HDR UK), London, UK; 2 Centre for Pharmacoepidemiology and Drug Safety, NIHR Greater Manchester Patient Safety Translational Research Centre, School of Health Sciences, Faculty of Biology, Medicine and Health, The University of Manchester, Manchester, UK; 3 Primary Care Research Centre, University of Southampton, Southampton, UK; 4 Primary Care and Public Health, Imperial College London, London, UK; 5 Institute for Global Health, Keele University, Keele, UK; 6 Department of Primary Care & Population Health, Institute of Epidemiology & Health, University College London, London, UK; 7 Centre for Academic Primary Care, University of Nottingham, Nottingham, UK; 8 Health eResearch Centre, University of Manchester, Manchester, UK; 9 Department of Health and Community Sciences (Medical School), Faculty of Health and Life Sciences, University of Exeter, Exeter, UK; 10 Clinical Effectiveness Group, Queen Mary University of London, London, UK; 11 Population Health Sciences, Bristol Medical School, University of Bristol, Bristol, UK; 12 Centre for Academic Primary Care, University of Bristol, Bristol, UK; 13 NIHR Bristol Biomedical Research Centre, Bristol, UK; 14 Health Data Research UK South-West, Bristol, UK; 15 NIHR Applied Research Collaboration (ARC) West, Bristol, UK; 16 Division of Informatics, Imaging and Data Sciences, University of Manchester, Manchester, UK

**Keywords:** electronic health records, primary care databases, population level linked data, population, primary health care

## Abstract

**Background:**

The range and scope of electronic health record (EHR) data assets in the UK has recently increased, which has been mainly in response to the COVID-19 pandemic. Summarising and comparing the large primary care resources will help researchers to choose the data resources most suited to their needs.

**Aim:**

To describe the current landscape of UK EHR databases and considerations of access and use of these resources relevant to researchers.

**Design & setting:**

Narrative review of EHR databases in the UK.

**Method:**

Information was collected from the Health Data Research Innovation Gateway, publicly available websites and other published data, and from key informants. The eligibility criteria were population-based open-access databases sampling EHRs across the whole population of one or more countries in the UK. Published database characteristics were extracted and summarised, and these were corroborated with resource providers. Results were synthesised narratively.

**Results:**

Nine large national primary care EHR data resources were identified and summarised. These resources are enhanced by linkage to other administrative data to a varying extent. Resources are mainly intended to support observational research, although some can support experimental studies. There is considerable overlap of populations covered. While all resources are accessible to bona fide researchers, access mechanisms, costs, timescales, and other considerations vary across databases.

**Conclusion:**

Researchers are currently able to access primary care EHR data from several sources. Choice of data resource is likely to be driven by project needs and access considerations. The landscape of data resources based on primary care EHRs in the UK continues to evolve.

## How this fits in

This narrative review is intended to provide an update on the continually evolving UK landscape of primary care EHR-linked databases available for research purposes. Similar reviews have been conducted previously; however, with the emergence of newer linked data assets, this update provides a current view of these different data assets, providing detail on scale, scope, and data sources within each, as well as how researchers can access them, costing models across each, and the training and accreditation required.

## Introduction

Information held in EHRs is a valuable research resource, particularly where the source data systems have near universal, longitudinal population coverage, as is the case with UK primary care EHRs. Given that the main purpose of EHRs is for clinical management, great care on interpretation is needed when data are used for research. Many issues of data completeness and quality, alongside the biases inherent in observational epidemiology, attach to analyses based on them; these are discussed below. This notwithstanding, EHRs have supported observational research for several decades.^1,2^

The range and scope of EHR-based data assets in the UK has recently increased, which has been primarily in response to the COVID-19 pandemic. Newer data assets may be less familiar to researchers, making their choice of the data resource most appropriate for their intended study difficult. This review aimed to summarise the current major sources of primary care EHRs data resources in the UK, alongside key characteristics of these relevant to potential users. It is hoped this information will help researchers choose the data resource most suited to their needs.

The review focused exclusively on UK EHR resources. Global resources, their development, and their uses are discussed elsewhere.^3,4^ Similarly, discussion of important issues, such as controversy around data sharing and patient perspectives, is beyond the scope of this article but these are discussed elsewhere.^5^

### Historical context

In the UK, primary medical care moved progressively from paper-based to electronic records from the late 1980s. Record-keeping in UK primary care is now almost exclusively electronic.^6^ A variety of commercially supplied clinical software systems are used in primary care. Currently, the following three vendors dominate the UK market: EMIS Health; SystmOne (provided by The Phoenix Partnership; TPP) and Vision (Cegedim Healthcare Solutions). Partnerships between practices, system vendors, academics, and for-profit companies subsequently made subsets of electronic primary care health records available for research.

These partnerships led to the formation of the General Practice Research Database now known as the Clinical Practice Research Datalink (CPRD),^7,8^ QResearch,^9^ The Health Improvement Network (THIN)^10^ database, and Optimum Patient Care Research Database (OPCRD).^11^ The Royal College of General Practitioners (RCGP) has supported practice-based infectious disease surveillance since 1957.^12^ This system is now electronic and supports a broader Research and Surveillance Centre (RCGP RSC).^13^ More recently, other partnerships have arisen (see below).

The population coverage of each database reflects the popularity and geographical reach of the parent systems^6^ as well as the practices that opt into them. EMIS Health is the most common provider to practices across the UK, and EMIS Health and TPP cover more than 90% of practices in England.

Initially, the major focus of EHR research was pharmaco-epidemiology but their research use now encompasses most aspects of observational epidemiology, including risk prediction,^14–17^ health services research,^18–20^ and clinical trials.^21^ This expansion has been facilitated by enhancement of EHR resources through linkage to other administrative data and to data collected in research studies and clinical audit.

### Whole population coverage

Statistical power in EHR research reflects sample size, whereas external validity is related to sample representativeness of the target population. Whole population coverage of an EHR database in a single nation has proved difficult to achieve for technical, socio-political, and legal reasons. Under the EU General Data Protection Regulation (GDPR) and the UK Data Protection Act (DPA) 2018, the legal data controller of primary care is the GP practice, which is responsible for the legal use of data and can decide whether data from practice patients may be processed for research purposes.^22^

Pre-COVID-19, Wales was the only UK nation to achieve near full-population coverage in a primary care EHR research database. The Welsh Longitudinal General Practice Dataset (WLGP),^23^ hosted by SAIL Databank,^24,25^ provides coverage of 83% of the population of Wales and 80% of Welsh GP practices. It is linked to other routine health and administrative datasets.^26^

### COVID-19 pandemic response

The COVID-19 pandemic created a situation where observational research based on EHR data at scale became a public health and policy priority, to identify risk factors for and sequelae of infection, and to investigate the effects of treatment and prevention measures. To enable this, a Notice under Regulation 3(4) of the Health Service (Control of Patient Information) Regulations 2002 (COPI) was introduced, covering England and Wales, by the Secretary of State for Health in March 2020, which directed general practices to provide primary care information deemed essential for the COVID-19 response.^27^

New EHR-based UK data resources have been enabled by the pandemic response, including a minimised primary care data extract, GP Data for Pandemic Planning and Research (GDPPR). A partnership between Health Data Research UK (HDR UK), NHS Digital, and the British Heart Foundation (BHF) formed the BHF Data Science Centre-led CVD-COVID-UK/COVID-IMPACT Consortium.^28^ This project resulted in the NHS Digital Trusted Research Environment (TRE), now NHS England Secure Data Environment (SDE); and enabled research relevant to COVID-19 with linkage to other datasets held by NHS England. The Consortium also includes other national TREs; SAIL Databank and the Scottish National Data Safe Haven.^29^ OpenSAFELY is a new TRE project created in collaboration across the Bennett Institute at the University of Oxford, the EHR Research Group at the London School of Hygiene and Tropical Medicine (LSHTM), the EHR suppliers TPP SystmOne and EMIS Health, and NHS England. The open source OpenSAFELY software tools are implemented inside the data centres of TPP and EMIS to enable secure and federated analysis of all structured GP data without the need for raw data to be extracted and disseminated.^30,31^

Other UK nations established large EHR-based data resources to support COVID-19-related research, including the Early Pandemic Evaluation and Enhanced Surveillance of COVID-19 (EAVE II) database in Scotland.^32^

Given this evolving landscape, the review aims to provide a summary and comparison of the current UK-based large primary care EHR data resources, as a guide to researchers.

## Method

The Health Data Research Innovation Gateway^33^ was searched with the term 'primary care'. This search was supplemented with information from key informants in the National Institute for Health and Care Research (NIHR) School for Primary Care Research^34^ and the wider primary care research community. Consideration was restricted to datasets openly accessible to external researchers.

### Sources of primary care data for research purposes

#### National resources

Nine data resources were identified, which are described in Supplementary Tables S1 and S2. Each includes patients resident in one or more of the UK nations. The summary characteristics tabulated were obtained via publicly accessible websites and published data. Data providers were contacted to confirm accuracy and completeness of information.

#### Regional data sources

Some UK regions have developed local EHR databases, with linkage to primary care data, to support care delivery and planning; NHS business intelligence; and research. Some of these resources are accessible to researchers, although this has been generally restricted, to date, to local analysts. Because of this, these resources are not described in detail. Examples include a regional network of TREs in Scotland^35^ such as DataLoch;^36^ and others across England such as Combined Intelligence for Population Health (CIPHA),^37^ HDR UK hub Discover-NOW,^38^ the Bristol, North Somerset and South Gloucestershire systemwide dataset,^39^ and the Connected Bradford database.^40^ Regional EHR data will eventually become more accessible for research through the current NHS England Data for Research & Development (R&D) Programme to develop an interoperable network of NHS-owned subnational SDEs across England.^41^

## Discussion

### National primary care EHR data resources

Researcher-relevant characteristics of the nine data resources identified are described below.

#### 1. Scope, scale, and data source

CPRD, QResearch, and THIN work with software suppliers to aggregate EHRs from practices that opt in. RCGP RSC and OPCRD hold agreements at the practice level to provide data and create resources that include records from different EHR vendors. Individuals can opt out of data sharing through contacting their practice.

OpenSAFELY provides secure access to full de-identified EHR records held by TPP and EMIS (>99% of patients in England, combined),^31^ and enables consistent, federated analysis across the two. A GDPPR extract is available from NHS England Data Access Request Service,^42^ in addition to access via the NHS England SDE. Use of these resources is currently enabled by COPI transitionary provision. General use beyond the pandemic is under negotiation.

These large data resources include records from between 3 and 70 million individuals with varying person follow-up time (see Supplementary Tables S1 and S2). Reported size of the data resource may include historic patients now deceased or embarked (that is, patients who have left the geographical catchment area of the resource) such that the number of live, registered patients may be lower than total numbers reported. For example, as of November 2022 CPRD reports 60 million patients, of which 18 million are currently registered active patients, with at least 20 years of follow-up for 25% of the patients.^43^ There is substantial overlap of patients represented between data resources.

All resources identified have been enhanced through linkage to other administrative data to a varying extent. Typically linkage is to secondary care records, death records, cancer registrations, and census-derived sociodemographic measures. More recently, linkage has been expanded to other datasets such as COVID-19 testing, immunisation, and intensive care.

Users typically must demonstrate a level of skills and experience appropriate to their intended research before gaining access, and may have to evidence completion of specific training, in addition to information governance and data security training.

In addition to supporting observational research, some resources offer extra research services; for example, to facilitate data-enabled trials.^21^

Refer to Supplementary Table S1: *Scope, scale and restrictions on use of UK primary care data resources*, for a detailed description of the scope, scale, and data sources of data assets.

#### Mechanisms of data access

##### Access models

Across these resources, data are accessed either through provision of a study-specific extract with assurances around security, appropriate handling, and data deletion or via a TRE or SDE. In both cases, the process typically involves several steps.

##### Steps and timescales

Typically, potential users are required to submit a proposal to an oversight committee. 'Access times' often describe time to this approval rather than time to data access, which can be misleading. Time to data access depends on multiple considerations that can incur considerable delays, these include the following:

- Ethical and other approvals: access to some resources requires prior ethical and R&D approvals to be in place. Some data resources have pre-approval from research ethics committees for particular types of research. Complex linked data applications and non-observational studies are more likely to require prior ethical approvals.
- Accreditation: this may be at the institutional or individual level. Some resources require organisations to have the NHS Data Security and Protection Toolkit^44^ in place, in line with GDPR. Individual users may be required to complete specific training such as Safe Researcher Training offered by the UK Data Service. Some resources provide training for main users of the data, with the expectation that knowledge is passed on within the user institute. Some resources do not specify particular training requirements but expect applicants to evidence specific competencies.
- Application process: beyond completion of an application form, the application process may necessitate engagement with the data provider to discuss the proposed research; for example, to estimate feasibility and statistical power. The more elaborate this process, the greater time required.
- Linked data: this typically requires additional permissions, causing delays particularly when the linkages sought are new rather than established. New linkages, where available, will generally incur greater costs and delays.
- Data preparation and processing: depending on the data resource and project, preparation of a suitable extract or pre-processing of data made available through a TRE or SDE may incur further delays.

### 2. Funding models for access

Data are made accessible through the following three main funding models: (a) an annual licence (some negotiated at an organisation level); (b) per project, which may include a base cost with additional charges representing resources in preparing bespoke or complex data requests or linkages; (c) on an academic collaboration basis.

Refer to Supplementary Table S2: *Access processes and requirements for primary care data resources*, for detailed description of data access mechanisms and processes across data assets in scope.

### 3. Analysis of primary care EHRs

Once data have been accessed as above, several considerations apply to the analysis process.

### Data wrangling and curation

Data wrangling and curation describe the processes of preparing the data before they can be analysed. The readiness of data for analysis varies depending on the data resource. Resources generally provide some form of data dictionary or data notes describing metadata and provenance of the data. Clinical and prescription data is commonly provided in a structured clinical vocabulary agnostic to the source system. Common formats include SNOMED CT (Systematized Nomenclature of Medicine Clinical Terms), Read Codes, ICD-10 (International Classification of Diseases, Tenth Revision) codes, as well as local codes (which may be less interoperable). Sometimes a combination of these is used.

The extent of curation needed varies with study design, but may include manipulating tables, deriving variables, linking data sources, and identifying study cohorts. Where several studies require similar manipulation of data, reusing a common code is helpful. Some resources require users to share code, using repositories such as GitHub.^45^ OpenSAFELY requires all code to be posted on GitHub before execution and publishes links to all executed code automatically at jobs.opensafely.org; analysts use standardised OpenSAFELY dataset building tools, which are integrated with the codelist development and sharing tools at OpenCodeLists.org.^46^

The CVD-COVID-UK/COVID IMPACT Consortium publishes protocols, code, and phenotype code lists via the HDR UK Gateway and GitHub.

Another common step required of analysts is to create EHR phenotypes that describe clinical concepts. Phenotype libraries and other resources to support standardisation and reproducibility have also been developed.^47–51^ Publishers may expect authors to provide code lists, algorithms, and programme files as supplements in published articles.

### Using a Trusted Research Environment (TRE) or Secure Data Environment (SDE)

Several data resources provide access via a TRE or SDE. Models vary in several ways, including the following:

- the prepackaged tools and software available in the analytical environment;
- the ability to import a user’s own code or software;
- availability of code for common data management tasks;
- the degree to which previous users’ data curation, variable derivation, and documentation is available to new users;
- threshold of small number suppression to protect against risk of patient reidentification;
- the level of user support available;
- ease of use;
- cost of use.

Some models allow curation, documentation, novel variable derivation, and associated documentation to be stored beyond the life of a single project or analysis and made available to future users, increasing the value of the resource. A UK Health Data Research Alliance White Paper^52^ has set out guidelines and principles for TRE and SDE good practice structured around the 'Five Safes' framework,^53^ and the Goldacre Review recommended use of TREs and SDE as the norm for analysis of health data.^54^

### Methodological and other considerations for working with primary care EHR data resources

#### Clinical context

Primary care EHRs are created primarily to support continuity in clinical care, as a medico-legal document, and to support payment systems. Their use in research needs to take into consideration why and how the data were collected. Because of this, experience of creating EHRs can help in guiding and interpreting analysis of them. Data recording and coding is influenced by many considerations. Understanding these, how they influence the content of the record, and the potential for bias to be introduced is essential to making valid inferences.^55^

#### Analytic and epidemiological considerations

Working with these data requires considerable epidemiological and analytical experience, including knowledge of common analytical tools and experience in handling large data resources. Access may be contingent on evidencing these competencies.

Population-level data also have characteristics that can make them challenging to use.^56^ Missing data and misclassification are key issues. Data are unlikely to be missing completely at random. Multiple imputation can be used to address this; however, it may introduce additional bias if used inappropriately.^57^ Sometimes missingness can be addressed through linkage to other data, facilitating the assessment of the extent of potential bias.^57^ Research questions must be evaluated for feasibility against the quality of the available data. For example, recording and management of many chronic conditions, risk markers, and other aspects of care have been incentivised in UK primary care, potentially introducing variations in data quality between information whose recording is or is not incentivised.^58^

Other epidemiological considerations are those attached to the difficulty of making valid causal inference in observational data where exposure allocation is non-random. The main issue is confounding by indication, where risk of exposure is associated with risk of outcome through a pathway independent of exposure.^59^ Collider bias^60^ and immortal time bias^61^ are also frequently important. The nature of causes, causal inference, and addressing bias attached to this endeavour have been discussed elsewhere, both in general terms^62^ and in the context of EHRs.^63^

### Future work and future developments

Models and mechanisms for accessing primary care EHRs, enhanced through linkage to other information, continue to evolve. This information is likely to include non-health administrative data, research data, patient-reported data, and data from patient-based and other sensors. Eventually this evolution may lead to near-whole population, real-time data from across the health and care system, linked to multimodal data from other sources being readily, securely, and acceptably available for analysis. Multiple biases will attach to these analyses and appreciation of their possible influence is important, particularly when analysis is genuinely intended to inform policy choices. Strategies to address these biases will also evolve. The broad term 'artificial intelligence' is currently applied to a variety of automated analytical approaches (including machine learning and deep learning) intended to make the extraction of useful inference from multimodal data more efficient and reliable.^64,65^ Linkage-enhanced data from health and care systems is likely to increasingly provide the substrate for such methods. Ultimately, this may lead to better understanding of the forces shaping human health and wellbeing, both in individuals and between social groups. This may support action to reduce inequities in these outcomes.

This article summarises major UK primary care data resources in terms of their strengths, weaknesses, and the opportunities they provide for researchers. Securing access to an appropriate dataset for research is often a complex transaction, for reasons described above. This article is intended to help researchers navigate that complexity. This is also a rapidly evolving landscape, shaped by multiple social, technical, and political considerations. In general, the trend is towards more streamlined, secure, and transparent access to better data, with the ambition that this will ultimately lead to health improvement for individuals and populations.
